# Quantifying the impact of regional variations in COVID-19 infections and hospitalizations across Ireland

**DOI:** 10.1093/eurpub/ckab173

**Published:** 2021-09-16

**Authors:** M Roe, P Wall, P Mallon, D Sundaram, J Kumawat, M Horgan

**Affiliations:** 1 School of Public Health, Physiotherapy & Sports Science, University College Dublin, Dublin, Ireland; 2 School of Medicine, University College Dublin, Dublin, Ireland; 3 Department of Infectious Diseases, St. Vincent’s University Hospital, Dublin, Ireland; 4 Department of Medicine, University College Cork, Cork, Ireland; 5 Infectious Diseases Clinic, Cork University Hospital, Cork, Ireland

## Abstract

**Background:**

As most COVID-19 transmission occurs locally, targeted measures where the likelihood of infection and hospitalization is highest may be a prudent risk management strategy. To date, in the Republic of Ireland, a regional comparison of COVID-19 cases and hospitalizations has not been completed. Here, we investigate (i) the variation in rates of confirmed infection and hospital admissions within geographical units of the Republic of Ireland and (ii) frequency of deviations in risk of infection or risk of hospitalization.

**Methods:**

We analyzed routinely collected, publicly available data available from the National Health Protection and Surveillance Centre and Health Service Executive from nine geographical units, known as Community Health Organization areas. The observational period included 206 14-day periods (1 September 2020–15 April 2021).

**Results:**

A total of 206 844 laboratory-confirmed cases and 7721 hospitalizations were reported. The national incidence of confirmed infections was 4508 [95% confidence interval (CI) 4489–4528] per 100 000 people. The risk of hospital admission among confirmed cases was 3.7% (95% CI 3.5–3.9). Across geographical units, the likelihood that rolling 14-day risk of infection or hospitalization exceeded national levels was 9–86% and 0–88%, respectively. In the most affected regions, we estimate this resulted in an excess of 15 180 infections and 1920 hospitalizations.

**Conclusions:**

Responses to future COVID-19 outbreaks should consider the risk and harm of infection posed to people living in specific regions. Given the recent surges of COVID-19 cases in Europe, every effort should be made to strengthen local surveillance and to tailor community-centred measures to control transmission.

## Introduction

Transmission dynamics driving the 2019 coronavirus (COVID-19) pandemic have been characterized by^1^ cross-species transmission,^2^ transfer of respiratory aerosols, especially in crowded or confined spaces,^3^ superspreader events (SSEs) where a small number of carriers contribute to a large number of secondary infections,^4^ emergence of community transmission from clusters linked with SSEs,^5^ mobility to/from outbreak sites, and^6^ selective adaptation in localized progenitor reservoirs which maintains the virus pool.^1–3^

As multiple factors influence each of these transmission characteristics, a large variation in how COVID-19 unfolds in different geographical regions is foreseeable. However, given the strain that the ongoing COVID-19 pandemic is placing on healthcare systems, it is important to identify whether people in specific regions of a country are at a disproportionate risk of acquiring infection or experiencing adverse outcomes. These insights are fundamental in designing infection control measures given that the same infection prevention strategy may not be equally effective in a diverse range of regions.^4^

Informed risk management requires the best available data to manage the probability of transmission, the probability of harm and the effectiveness of mitigating measures.^5^ As these metrics are anchored in the fundamentals of risk management, they may be more informative than commonly used epidemiological metrics, such as the reproduction number which hides the complexities of COVID-19 transmission being highly stochastic with low population prevalence.^6^ Such attempts to regress rates of secondary infection in multiple areas to a national mean, overlooks contextual factors within geographical units that influences known transmissions characteristics likely extending chains of transmission.^7^

Therefore, the aim of this study is to investigate the variation in rates of confirmed infection and hospital admissions within geographical units of the Republic of Ireland. Additionally, we sought to investigate the frequency at which specific geographical units experienced large deviations in risk of infection or risk of hospitalization.

## Methods

We used observational, routinely collected, publicly available data available from two national surveillance systems in the Republic of Ireland. First, 14-day epidemiology reports of COVID-19 were published by Health Protection and Surveillance Centre (HPSC). Second, COVID-19 Daily Operations Updates published by the Health Service Executive (HSE), Ireland’s national public health and social care services provider. These services are delivered across nine geographical units known as Community Health Organization (CHO) areas.

### National surveillance systems

The HPSC report data recorded on the national Computerized Infectious Disease Reporting system, which registers each laboratory-confirmed infection as detected on real-time polymerase chain reaction testing of oropharyngeal and nasopharyngeal swabs. These HPSC reports provided the number of laboratory-confirmed cases of COVID-19 and population in each CHO area. The HSE reports provided the number of confirmed COVID-19 cases among patients in each acute public hospital in the Republic of Ireland over the prior 24-h period as of 8 pm on the date of reporting.

### Data extraction

We extracted data from publicly available HPSC and HSE reports for dates ranging from 1 September 2020 to 15 April 2021 (first national case confirmed 29 February 2020) to an Excel file (Microsoft, Seattle).^8^^,^^9^ These dates coincided with the first public reporting of confirmed cases with each CHO geographical unit in the prior 14 days. Across the 227-day observational period, 21 periods were excluded due to incomplete data. This resulted in 206 14-day periods being included.

Extracted data were processed in the following ways. First, individual hospital-level data from HSE reports were assigned to a corresponding CHO area. This provided a sum number of the total number of confirmed COVID-19 cases among hospital inpatients per geographical unit in the prior 24 h. Second, we used this data to compute a rolling 14-day total number of confirmed cases among hospital inpatients per geographical unit.

### Data analysis

We calculated the incidence per 100 000 of laboratory-confirmed infections per geographical unit using the CHO area population as a denominator. We also calculated the absolute risk of hospital admission per geographical unit using the number of confirmed cases per CHO area as a denominator, and number of confirmed cases among hospital inpatients per CHO area as the numerator.

Relative risk (RR) was calculated to compare the (i) risk of infection and (ii) risk of hospital admission in each CHO area relative to all other geographical units. The frequency at which RR was statistically significantly higher (*P* ≤ 0.05) than all other geographical units was calculated for each CHO area.

Excess infections and hospital admissions per geographical unit were calculated as the absolute difference between reported events and expected events as estimated by a standardized incidence ratio (SIR). The SIR compared the event rate of each CHO area to all other geographical units.

Using the national average across all observations, we calculated a standardized or *z*-score for the (i) risk of infection and (ii) risk of hospitalization for each 14-day period. Previously, a *z*-score of 2 (i.e. the value is two standard deviations above the mean) has been used as cut-off point to monitor deviations from expected levels of mortality in Europe.^10^ The frequency at which *z*-scores ≥2 and ≥3 was calculated for each geographical unit.

We also calculated 95% confidence intervals (95% CIs) to estimate the precision of these measures. Data were analyzed in MS Excel (Microsoft, V16.49). Figures were generated in Tableau (V2020.4.5). Results are presented following a review of REporting of studies Conducted using Observational Routinely-collected Data guidelines.^11^

### Ethical statement

Institutional review board approval was not sought for this analysis of publicly available, deidentified data.

## Results

A total of 206 844 laboratory-confirmed cases and 7721 hospital inpatients with COVID-19 were reported during the observational period. The national incidence of confirmed infections was 4508 (95% CI 4489–4528) per 100 000 people. The risk of hospital admission among confirmed cases was 3.7% (95% CI 3.5–3.9). Table 1 outlines variations in incidence and hospital admission risk per geographical unit.

**Table 1:** Summary of statistics for average 14-day period observed per geographic unit from 1 September 2020 to 5 April 2021: incidence and hospital admission risk

	Incidence per 100 000	Incidence rate ratio	Risk of hospital admission	Risk ratio	Excess infections	Excess hospital admissions	Frequency exceeding national infection rate	Frequency exceeding national hospitalization rate
CHO 1	5004.0 (*n* = 19 468)	1.12	4.3%	1.16	2108	191	52.9%	32.0%
		(1.11–1.14)	(*n* = 829)	1.08–1.24			(*n* = 109)	(*n* = 66)
CHO 2	3784.3 (*n* = 16 854)	0.83	4.8%	1.33	−3570	75	9.2%	28.3%
		(0.81–0.84)	(*n* = 816)	(1.25–1.43)			(*n* = 19)	(*n* = 54)
CHO 3	4333.1 (*n* = 16 437)	0.96	1.4%	0.36	−724	−443	23.3%	0.0%
		(0.94–0.97)	(*n* = 231)	(0.31–0.41)			(*n* = 48)	(*n* = 0)
CHO 4	3603.9 (*n* = 23 949)	0.77	2.3%	0.59	−7027	−655	12.1%	0.0%
		(0.76–0.78)	(*n* = 557)	(0.55–0.65)			(*n* = 25)	(*n* = 0)
CHO 5	3931.8 (*n* = 19 564)	0.86	4.7%	1.30	−3216	96	18.4%	39.8%
		(0.85–0.87)	(*n* = 922)	(1.22–1.38)			(*n* = 38)	(*n* = 82)
CHO 6	4618.1 (*n* = 16 831)	1.03	2.6%	0.67	435	−196	36.4%	7.3%
		(1.01–1.04)	(*n* = 432)	(0.61–0.74)			(*n* = 75)	(*n* = 15)
CHO 7	4419.1 (*n* = 29 788)	0.98	2.0%	0.49	−703	−638	25.2%	6.3%
		(0.97–0.99)	(*n* = 589)	(0.45–0.53)			(*n* = 52)	(*n* = 13)
CHO 8	4689.9 (*n* = 27 783)	1.05	4.1%	1.09	1237	135	53.4%	21.8%
		(1.03–1.06)	(*n* = 1113)	(1.03 - 1.15)			(*n* = 110)	(*n* = 45)
CHO 9	6220.4 (*n* = 36 171)	1.46	6.1%	1.91	11 402	1426	86.4%	88.3%
		(1.44–1.48)	(*n* = 2223)	(1.84–1.99)			*n* = 178	(*n* = 182)

Geographical units are classified at Community Health Organization (CHO) level. CHO 1 includes Donegal, Monaghan, Cavan, Leitrim, Sligo; CHO 2 includes Roscommon, Mayo, Galway; CHO 3 includes Clare, Limerick, North Tipperary; CHO 4 includes Kerry, Cork; CHO 5 includes South Tipperary, Waterford, Carlow, Kilkenny, Wexford; CHO 6 includes Dublin South East, Dun Laoighaire, Wicklow; CHO 7 includes Dublin South City, Dublin South West, Dublin West, Kildare, West Wicklow; CHO 8 includes Laois, Longford, Offaly, Westmeath, Meath, Louth; CHO 9 includes Dublin North, Dublin North Central, Dublin North West. Data presented with corresponding 95% CIs. Rate and risk ratios refer to comparison to all other geographical units. Excess estimates refer to absolute difference relative to expected levels using standardized incidence ratio for all other geographical units. Frequency exceeding national rates refers to number of times prior 14-day period was statistically significant (*P* ≤ 0.05) higher than national levels.

Based on the expected number of events as determined from SIR calculations, we estimate a range of excess confirmed infections across the nine geographical of −3570 to +11 402. Similarly, the range of excess hospital admissions was −655 to +1426 across the nine geographical units (table 1).

**Figure 1:**
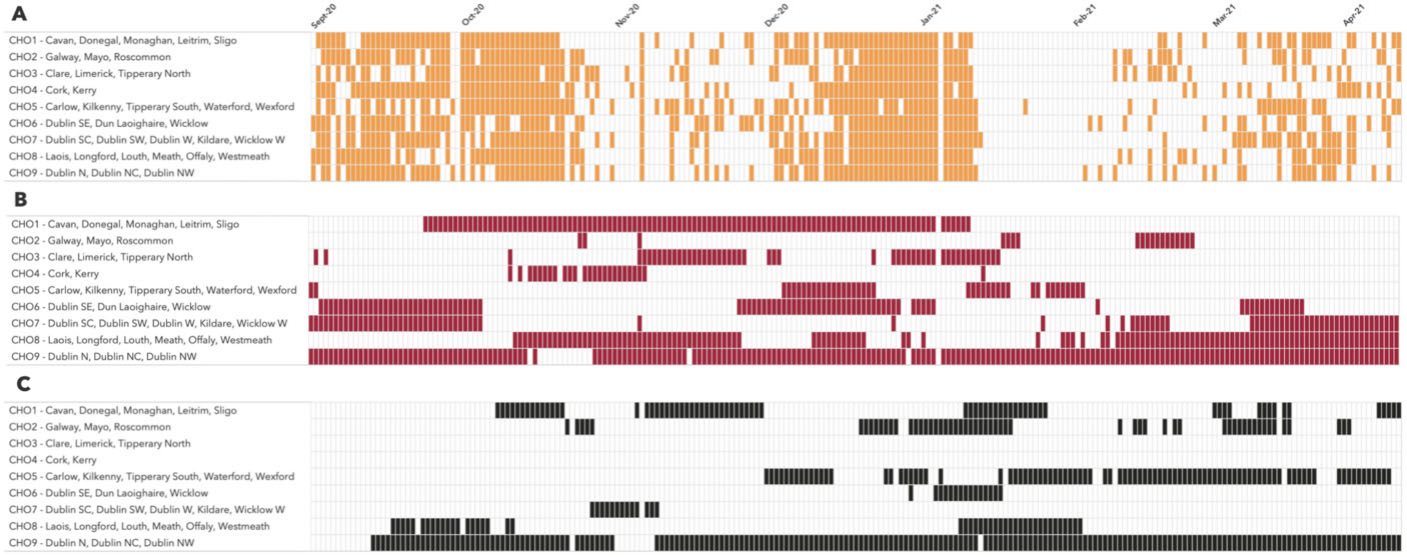
Outline of 14-day periods from 1 September 2020 to 15 April 2021 when (A) laboratory-confirmed cases increased relative to previous 14-day period, (B) incidence of confirmed infection was significantly higher (*P* ≤ 0.05) than all other regions and (C) risk of hospital admission in confirmed cases was significantly higher (*P* ≤ 0.05) than all other regions

The frequency at which RR of confirmed infection was statistically significantly higher (*P* ≤ 0.05) than all other geographical units ranged from 9.2% (*n* = 25 days) to 86.4% (*n* = 178 days) (figure 1). The frequency of statistically significant higher RR of hospital admission also varied between geographical units, ranging from 0.0% (*n* = 0 days) to 88.3% (*n* = 182 days).

The frequency of large deviations, as defined by *z*-scores ≥2 and ≥3, in confirmed infections and hospital admissions per geographical unit in the prior 14-day period are presented in table 2. These indicate a difference between geographical units in the frequency of incidence of confirmed infection for *z*-score ≥2 (range = 6.3–39.3%) but not *z*-score ≥3 (range = 0.0–35.4%). We also observed large differences in the variation between geographical units for the frequency by which risk of hospital admissions deviated from the national average indicated by a *z*-score ≥2 (range = 0.0–54.4%) and *z*-score ≥3 (range = 0.0–35.9%).

**Table 2:** Frequency of deviations from national levels during observed 14-day periods from 1 September 2020 to 15 April 2021

	Frequency	Frequency	Frequency	Frequency
	incidence of	incidence of	risk of	risk of
	confirmed infection	confirmed infection	hospital	hospital
	≥2*z*	≥3*z*	admission	admission
			≥2*z*	≥3*z*
CHO 1	39.3% (*n* = 81)	35.4% (*n* = 73)	19.4% (*n* = 40)	9.7% (*n* = 20)
CHO 2	6.3% (*n* = 13)	0.0% (*n* = 0)	13.1% (*n* = 27)	0.5% (*n* = 1)
CHO 3	7.8% (*n* = 16)	5.3% (*n* = 11)	0.0% (*n* = 0)	0.0% (*n* = 0)
CHO 4	6.3% (*n* = 13)	4.4% (*n* = 9)	0.0% (*n* = 0)	0.0% (*n* = 0)
CHO 5	6.8% (*n* = 14)	4.9% (*n* = 10)	36.9% (*n* = 76)	20.4% (*n* = 42)
CHO 6	7.3% (*n* = 15)	4.4% (*n* = 9)	0.0% (*n* = 0)	0.0% (*n* = 0)
CHO 7	7.3% (*n* = 15)	3.4% (*n* = 7)	0.0% (*n* = 0)	0.0% (*n* = 0)
CHO 8	6.3% (*n* = 13)	1.5% (*n* = 3)	6.8% (*n* = 14)	0.0% (*n* = 0)
CHO 9	10.2% (*n* = 21)	6.3% (*n* = 13)	54.4% (*n* = 112)	35.9% (*n* = 74)

Geographical units are classified at Community Health Organization (CHO) level. CHO 1 includes Donegal, Monaghan, Cavan, Leitrim, Sligo; CHO 2 includes Roscommon, Mayo, Galway; CHO 3 includes Clare, Limerick, North Tipperary; CHO 4 includes Kerry, Cork; CHO 5 includes South Tipperary, Waterford, Carlow, Kilkenny, Wexford; CHO 6 includes Dublin South East, Dun Laoighaire, Wicklow; CHO 7 includes Dublin South City, Dublin South West, Dublin West, Kildare, West Wicklow; CHO 8 includes Laois, Longford, Offaly, Westmeath, Meath, Louth; CHO 9 includes Dublin North, Dublin North Central, Dublin North West.

## Discussion

This study investigated the variation in rates of (i) confirmed infection, (ii) hospital admissions and (iii) large deviations of infections and hospital admissions within geographical units of the Republic of Ireland. Collectively, these findings highlight the need to respond to the heterogeneity of infection levels within a country.

Incidence of confirmed infection was statistically significantly higher in two geographical units (CHO 1 and CHO 9). Elevated infections rates were recorded in these areas on 53–86% of observed 14-day periods. We estimate that this resulted in an excess of 13 500 infections. However, we note the higher risk of infection in the latter stages of the observational period in each CHO 6–9, which surround the capital city, locally known as the ‘commuter belt region’. Reducing risk of transmission in local areas requires a three-pronged approach involving surveillance, early detection and targeted outbreak management.^12^ These three elements provide insights on the variation of how health emergencies unfold in different geographical regions, directing the creation of bespoke measures to address how COVID-19 evolves and impacts communities living and working in certain parts of a country.

Data from Brazil illustrates two COVID-19 transmission patterns driven by transport from urban areas: first, from metropolitan areas to deeper inner state regions, and second, from urban areas of regional importance to less connected municipalities.^13^ Similarly, proximity to major transport hubs (e.g. international airports and national rail stations) has been associated with emergence of COVID-19 hotspots in India: as Gupta et al.^14^ outline, in the context of zoonotic diseases, ‘the problem with cities is that they create ecosystems of their own’ that extend transmission and prolong outbreaks, thereby amplifying case numbers. These urban travel dynamics have previously been cited as contributors to the rapid spread of early outbreaks from Wuhan to mainland China and Europe.^15^ Thus, preparedness for future health emergencies must account for (i) co-ordinated responses across multiple bureaucratic entities that govern countries, cities and communities and (ii) implementation of bespoke infection control measures that consider the forces shaping local communities.

We also found hospitalization risk to be heterogeneous across geographical units ranging from 1.4% to 6.1%. This indicates that the consequences of infection are not uniform in all geographical units and is likely attributable to the underlying population demographics within each area. Given the risk of adverse outcomes associated with COVID-19 and non-communicable diseases, the ‘the conditions in which people are born, grow, live, work and age’, need to be appraised.^16^^,^^17^ Future studies should profile local areas capturing population dynamics, characteristics of the built environment, such as housing and outdoor spaces, and underlying health conditions prevalent in local populations. This could function as a triage system that can assist local area planning and preparation for future health emergencies.

We also found that compared to national levels, deviations in infection and hospitalization rates associated with *z*-scores ≥2 and ≥3 exclusively occurred in three geographical units (CHO 1, CHO 5 and CHO 9). For instance, the likelihood of these areas experiencing increased hospitalization rates in prior 14-day periods, when case numbers were standardized, above 3SDs of the national average ranged from 10% to 36%. Periodically, this may result in excessive demands on healthcare facilities and workers in specific areas, particularly in resource-limited settings.^18^

A limitation of this study is our inability to determine the cause of elevated hospitalization risk in specific geographic units. We hypothesis two sources. First, a higher prevalence of underlying clinical conditions within ageing communities. Second, outbreaks on hospital wards leading to patients acquiring COVID-19 in healthcare facilities within each geographical unit. These two hypotheses warrant immediate investigation given the associated probability of harm. However, this limitation should not distract from the main findings of this study: the variation in risk of confirmed infection and hospital admission with COVID-19 across geographical units of the Republic of Ireland is profound yet consistent. This illustrates the need to design targeted infection prevention and control measures that offer protection to people within specific areas based on consideration of local contextual factors.

The heterogeneous distribution of COVID-related health events, across even relatively small European jurisdictions, highlights the need for real-time local-level surveillance to identify specific areas where risk is disproportionately higher.^19^ This should become an imperative given the surge in COVID-19 cases across Europe and urgency to prepare for future pandemics.

## Funding 

M.R. is funded by Science Foundation Ireland (SFI) through the COVID-19 Rapid Response Research and Innovation Programme.

*Conflicts* *of interest*: None declared.

**Key points**

- Over a 206-day observational period, we estimate that the number of excess confirmed infections ranged from −3570 to +11400 across nine geographical regions of Ireland. Similarly, the range of excess hospital admissions was −650 to +1400 across the nine geographical units.
- Relative to national levels, geographical regions of Ireland also experience surges in infection and hospitalizations rates at vastly different frequencies.
- As most COVID-19 transmission occurs locally, targeting infection control measures at areas where the likelihood of infection and hospitalization is greatest remains the most prudent risk management strategy.
- Strengthening the capacity for real-time local-level surveillance will assist the creation of community-centred measures that reduce transmission.
